# Perceived water insecurity among adults from urban and peri-urban Haiti: A qualitative study

**DOI:** 10.1371/journal.pone.0214790

**Published:** 2019-04-24

**Authors:** Elizabeth A. Wood, Hannah Douglas, Andrew J. Fiore, Meredith K. Nappy, Robinson Bernier, Kelly S. Chapman

**Affiliations:** 1 Department of Environmental & Global Health College of Public Health and Health Professions University of Florida, Gainesville, FL, United States of America; 2 College of Public Health and Health Professions University of Florida, Gainesville, FL, United States of America; 3 Department of Anthropology University of Florida, Gainesville, FL, United States of America; Anglia Ruskin University, UNITED KINGDOM

## Abstract

Water and sanitation services are fundamental in preventing the spread of waterborne and hygiene-related diseases. However, in developing countries, such as Haiti, access to clean water continues to pose major challenges despite efforts to improve quality and reduce distance. With Léogâne being the epicenter of the earthquake in Haiti in 2010, there were dozens of interventions aimed to improve access to clean water, specifically well construction and use of water treatment strategies. Using the socioecological framework, this study collected qualitative data to supplement a household water insecurity experiences (HWISE) survey in order to fully understand the narratives around water in Léogâne (urban) and its neighboring commune Gressier (peri-urban). The inclusion criteria for this study was that the participant must be a resident of either site, at least 18 years or older, and a female. Only females were included in this study so that researchers could better understand how perceived water insecurity impacts reproductive health, specifically gynecological infections. This cross-sectional study yielded 61 total in-depth interviews using a semi-structured open ended questionnaire to allow participants the ability to elaborate. Results suggest that there are common misconceptions about water and reproductive health specifically that engaging in sexual intercourse in saltwater will not result in pregnancy. Relevant narratives among the two communes included water acquisition, use of water, and bathing practices, among several others. Through understanding the local Haitian perspective and practices that surround water insecurity, we can better tailor public health interventions to improve access to water, female hygiene practices, and ultimately lower and prevent disease transmission.

## Introduction

Water insecurity has largely been conceptualized as a result of insufficient “access to and/or supply of” water for daily living [1–3]. The Joint Monitoring Program [4] defines improved drinking water sources as those that by nature of their design have the potential to provide safe water. The lack of access and/or availability of water is a burgeoning global health issue that can have serious health concerns. Through poor sanitation and hygiene (intensified by lack of safe water) there is an increased risk of spreading pathogenic microbes that increase morbidity and mortality [5]. Additionally, there are several studies that have already established that water insecurity, as it relates to access, safety, and financial barriers, is disproportionally found among poor, vulnerable populations [1, 6–8].

Improved water sources are still not sufficiently available for the majority of Haiti’s population, which significantly increases the risk of poor health outcomes including malnutrition and/or delayed physical, psychological, and cognitive development in children [5, 9–10]. Rural areas in Haiti have considerably less access to improved water sources compared to urban areas which contributes to disparities in overall health status [11]. Moreover, after the 7.0 magnitude earthquake in 2010, water accessibility, availability, and quality decreased further [10]. The cholera endemic that followed the earthquake exacerbated systems that were already fragile, pushing Haiti into a state of emergency that would trigger a flood of foreign aid [9]. Due to a lack of government communication, preparedness, and general oversight, foreign aid that was earmarked for rebuilding the country after the earthquake was not spent befittingly [12]. Consequently, Haiti still lacks the infrastructure and government capacity needed to support improved water sources, modify behaviors around poor hygiene, and design an adequate sanitation system for its inhabitants.

This study looks to investigate water insecurity in the Southern Peninsula of Haiti, in communes Gressier and Léogâne. A primary objective of this research is to examine the perceived heterogeneity between the communes (one being peri-urban and the other urban) in relation to water access, utility, and hygiene as it relates to water. This study aims to identify the current knowledge, attitudes, and practices that surround water insecurity in order to contribute literature on culturally grounded perceptions by local Haitians.

## Methods

### Study setting

**Fig 1:**
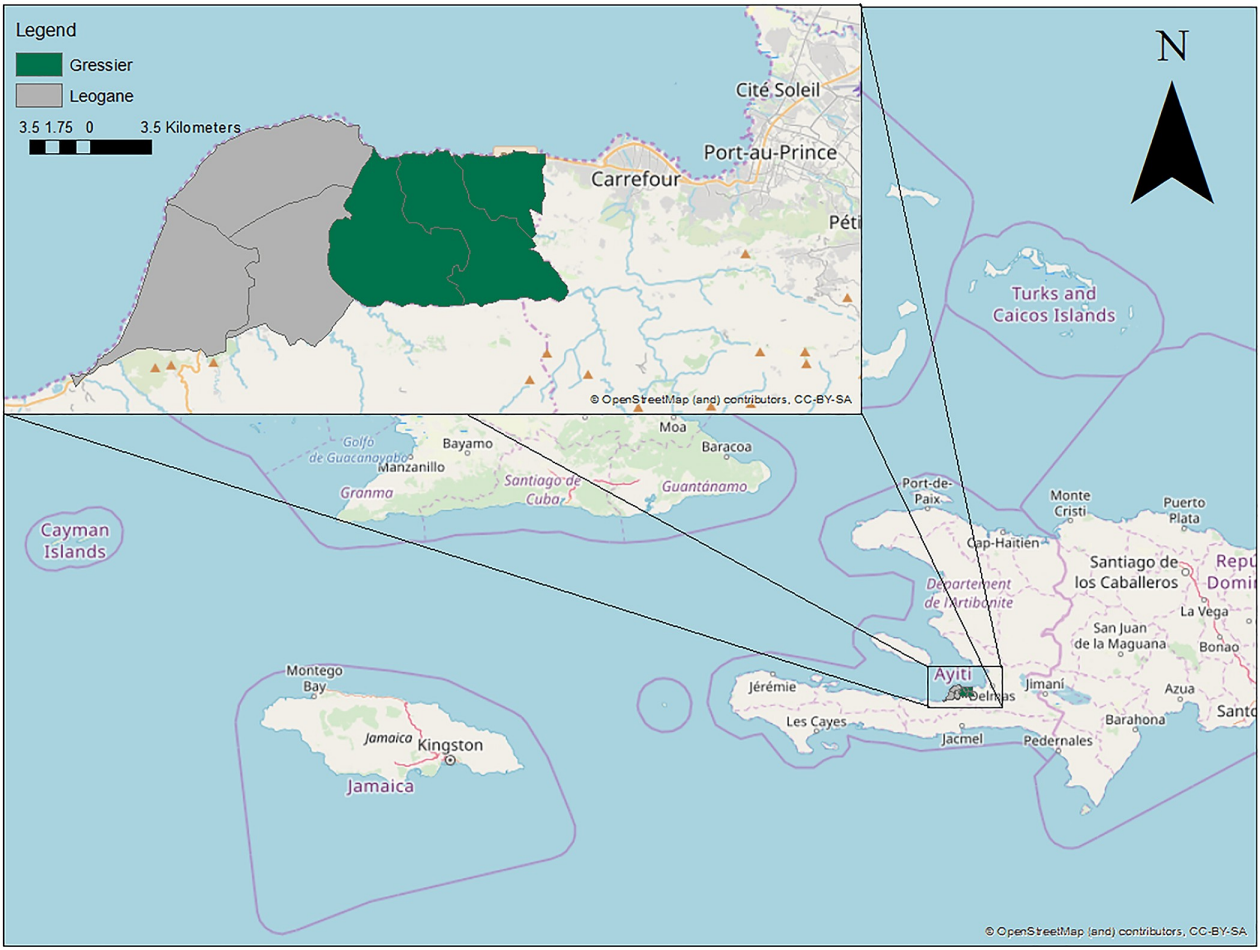
Catchment Area: Léogâne and Gressier, Haiti.

Gressier and Léogâne are two communes in the Ouest Department of Haiti that are side-by-side geographically and were selected because of their potential differences due to population size and land use as shown in Fig 1. Additionally, in 2010 Léogâne was the epicenter of the earthquake and has had substantial foreign and domestic aid to build better access to safe water. The University of Florida (UF) has also had a historical presence in the region with a there being a biosafety level-two and biosafety level-three public health laboratory located in Gressier since 2011 [13].

### Study design & data collection

From March to May 2018, UF researchers and students conducted a cross-sectional household water insecurity experiences (HWISE) survey to create a baseline in Gressier and Léogâne. This study built upon the HWISE survey and administered a qualitative component that this paper will address specifically. Researchers used a sequential explanatory design [14] for collecting the qualitative data in this study; wherein participants that were knowledgeable about water practices and utility from the HWISE survey were re-contacted for in-depth interviews. This framework was chosen to delve deeper into water insecurity questions from the survey that could explain a local phenomenon.

Fieldwork for this study began in March 2018 with UF students, including a PhD student in anthropology as well as a small group of eight undergraduate students with varying health and biological majors, assisting researchers and local Haitian staff. The undergraduate students only performed fieldwork in the second site, Léogâne. Prior to collecting data, all UF students were trained in qualitative data collection methods, which included but were not limited to, cultural sensitivity and competence among participants. A team of three local Haitian staff that have worked previously with UF researchers were employed as translators for the qualitative data collection component and were among the enumerators previously employed for the HWISE survey component. This team was trained previously by the UF researchers in qualitative data collection methods and had demonstrated competence in facilitating focus groups, conducting interviews, enumeration that included open-ended questions, best practices in avoiding bias, and community engagement and recruitment strategies.

Following the data collection of the HWISE surveys, selected participants were re-contacted (with permission) and asked to participate in in-depth interviews. These interviews served to contextualize themes from the HWISE survey and develop an understanding of how water insecurity was perceived within Haitian culture as well as to serve as the empirical foundation of the qualitative analyses in this paper [14]. All interviews were conducted by two UF students at a time with the help of the local translators that were present to communicate with the local participant and field questions between the two parties. While one UF student would ask questions and probe, the second acted as a scribe sitting beside or in close proximity to the Haitian translator. This was done to gather real-time translations of interviews from Haitian–Creole to English. Interviews were also recorded, with permission of all participants, and later reviewed by one of the Haitian staff to ensure quality within the English translation.

Inclusion criteria for this study required being 18 years of age or older, female, Haitian, and living in either Gressier or Léogâne. Only females were chosen for this study in order to ask targeted questions around reproductive health and hygiene practices, specifically regarding the transmission of bacterial or viral diseases. Exclusion criteria included any female that had not lived in the household for at least six months or was not knowledgeable about their current water practices. Interviews began in Gressier and were more fluid, with semi-structured questions being manifested from the participant’s specific HWISE survey responses. In doing this, a question bank was established that would be used during the Léogâne interviews. This study only looks to compare questions that were asked in both locations. Interviews were translated in real-time into the participant’s native language with the assistance of a local Haitian translator and lasted no more than two hours per participant. Questions were semi-structured to allow the participant the opportunity to expand on themes not covered by the HWISE survey. Sample size for the in-depth interviews was based on reaching saturation within the priority population, specifically in relation to knowledge, beliefs, and practices around water [15].

### Data analysis

Qualitative analyses were conducted on the in-depth interview transcriptions. Ethnographic content analysis, a method used to analyze qualitative data as a means to explore themes, was applied while coding the data [16]. Two UF researchers independently coded themes and subthemes iteratively based on manifest content, which resulted in a coding scheme with eight prominent categories. Certain themes were collected a priori given the content of the interviews was focused around content initially derived from the HWISE survey. Intra-rater reliability was established wherein each coder independently created a codebook, compared it with the other, and discussed each theme and subtheme with a third party until consensus was made to ensure validity. QSR International’s NVivo 12 software was used during this process to finalize salient themes and subthemes to demonstrate consistency among coders. Once each theme and subtheme were identified, they were defined as each related to water insecurity and compared by location. Trustworthiness and scientific rigor are established through the triangulation of data from the quantitative and qualitative knowledge obtained, reliability and validity of the thematic codebook, and using a flexible, iterative process to generate salient themes and subthemes.

### Ethics

The UF Institutional Review Board approved this study October 31, 2017 (IRB201702549). The Haitian Ministry of Health ethics review board approved this study on February 22, 2018. Data collection began once both approvals were obtained. Prior to recruiting any participant for this study, oral consent in Haitian-Creole was obtained. Oral consent was preferred in previous studies in this region of Haiti as a result of the low literacy rates [17–18].

## Results

There were a total of 499 surveys from both Léogâne and Gressier; with 32 participants from Gressier and 29 from Léogâne being re-contacted for the in-depth interviews. Table 1 provides sociodemographic characteristics of those that were interviewed with most of the sample being homogenous with the exception of those in Gressier receiving more health education than those in Léogâne. Themes that were discussed from the Léogâne and Gressier interviews included: (1) Water acquisition, (2) Damaged water source, (3) Community involvement, (4) Treatment, (5) Water separation & uses, (6) Bathing practices, and (7) Pregnancy risks.

**Table 1:** Socio-demographic characteristics for In-Depth Interview participants in Gressier & Léogâne.

	n (%)		
	Gressier 32 (57.1)	Léogâne 24 (42.8)
Socio-demographic characteristics			
Age		
Range	(16, 68)	(18, 66)
Mean (SD)	35.0 (13.8)	36.2 (12.5)
Median	33	35
Household size		
Range	(1, 10)	(2, 10)
Mean (SD)	4.7 (2.6)	5.5 (2.4)
Median	4	5
Median rated ladder run		
Income	9	8
Education	5	5
Safe work	9	8
Safe water	5	7
House type–n (%)		
Owned house	28 (87.5)	20 (83.3)
Rented house	4 (12.5)	4 (16.7)
Occupation–n (%)		
Trade/commerce	8 (25.0)	11 (45.8)
Education	4 (12.5)	3 (12.5)
Food service	3 (9.4)	0 (0.0)
Homemaker/supported	2 (6.3)	1 (4.2)
Nothing	11 (34.4)	5 (20.8)
Other	4 (12.5)	4 (16.7)
Works in–n (%)		
NGO sector	2 (6.3)	5 (20.8)
Healthcare sector	5 (15.6)	3 (12.5)
Received education–n (%		
About health	20 (62.5)	9 (37.5)
From work	7 (21.9)	6 (25.0)

In Léogâne, themes around water access were primarily related to a shortage of clean, safe water in the area. Due to this perceived shortage, water uses were largely determined by source. For example, it was explained in the interviews that well water was boiled and used for cooking, it was not to be used as a primary drinking source or bathing. There are many non-governmental organizations (NGOs) and international organizations in Léogâne that may contribute to why so many participants shared that they treat the water with aquatabs, tablets for disinfection of drinking water for human consumption. Alternatively, in Gressier, since the reported primary source of drinking water is different than Léogâne, participants described similar problems with access but in regard to piping systems instead of wells. Perceptions on bathing practices and pregnancy risks related to water were explored between the two communes as a result of previous work in the area around vaginal illness and water quality [18].

### Water acquisition

Issues related to accessing water was consistent across the two communes. Many participants explained that they choose to go to the water source that is closest to them out of necessity, however, it is usually not their preferred water source. Additionally, if the main water source was a well, participants in both communes acknowledged their frustrations with having a schedule around when the well would be unlocked and available for use. As quoted in Table 2, having locks on the wells can result in an excessive amount of people gathering at the well at once causing long lines that lead to hours of waiting or sometimes fights. The rules around each well differ superfluously, even between neighborhoods, however, as indicated by participants it seems unsystematic and regulated.

**Table 2:** Comparative Themes around Water Insecurity in Gressier and Léogâne.

Themes	Gressier	Léogâne
**Water Acquisition**	“If [I] cannot get water from the main source with the reservoir, then I will use the captured rainwater, because there isn’t another source close to me. [I] would have to go all the way to the national road for the next source.” “The water has a schedule, they have water in the morning, but it stops at noon and then it comes again in the afternoon around 4 or 5pm, this is every day. Sometimes the water comes in the afternoon and in the morning, sometimes they don’t shut off the source.”	“One thing that I don’t like is that sometimes you may be at water source for hours, and you can’t get water and sometimes you might get there, and it’s already closed. It doesn’t always open when it should.” “There are too many people for the water sources. For the people around, there are not enough water sources. We need more.” “When I cannot access it, it’s when it’s very crowded. And also, they close it if people are fighting, if they don’t behave well.”
**Damaged Water Source**	“The pipes used to work but now they aren’t working at all, for the past 3 months.” “They don’t work, and no one fixed them. They installed pumps on top of the well and you have to pump it, but the water doesn’t come now.” “[We] try to spend money on getting a pipe for water, we have a pipe in the yard now, but it’s doesn’t work. They spent their money, but no water comes.”	“People are coming around and breaking the head of the water source (the water capture flows from across the street to her yard) and it is not coming into their yard and they have to go to main water source to get water from there.” “When the pump has a problem or it’s broken, we have to cross National [highway] #2 to get to the water company. I don’t like to go back and forth. We have to cross the street and go to the water company.”
**Community Involvement**	“[They] had to come together as a community with the water committee to buy a new pipe/hose to fix the problem. The committee had people that were trained and could fix the problem.” “The people in the community all give a little bit of money for it, then if there is a problem they can fix it, they will have enough money for the repair. Just a very little amount of money.” “It’s a community pump, everybody looks after it. For example, if you see children pumping the water to hard or you hear a lot of noise, everyone nearby will stop them.”	“When there is a problem with the pump, everyone gets together and puts their money together to fix it.” “Yeah, the only one that we have access to. Everybody has access to this one, so when it has a problem, we all contribute.” “The community collects money to fix it when it is broken. No one maintains it, [we] just collect money all together and call someone to fix it.”
**Treatment**	“The only problem that we have is that [water] isn’t treated. We have the water, we get it for free from the ground, but ITECA (Institute of Technology and Animation) goes to get the water from other places and give you a pipe to get the water. We have a water source but it is not treated. It would be better if they had help to treat the water.” “They (organizations) don’t tell you how to treat [water], they just say to use treated water. I use Aquatabs and Gadyen Dlo, this is a liquid that they sell at the pharmacy to treat the water.” “I don’t treat the water that I store in the barrel, I use it to do laundry and to bathe. But if I want to use it to do other stuff, I will take it from the barrel and then treat it before using it.”	“We usually treat all of the water. We store water in barrels and treat that, but only if we have treatment. If there’s not enough to treat the barrel, they only treat what they take out to drink.” “We treat the water from the well when we use it, but the water from the pump is already treated. [We] treat the water before [we] bathe. “We usually treat all of the water. We store water in barrels and treat that, but only if we have treatment. If there’s not enough to treat the barrel, we only treat what we take out to drink.”
**Water Separation & Uses**	“[We] have different containers, drinking water is stored in Culligan gallons. [I] have a bucket for the kitchen and other buckets for bathing.” “[We] have different containers to store water in the house. The water that we use for laundry, bathing, and to do household chores is stored in barrels. The cooking water is stored in buckets with covers. And they have a blue Culligan gallon (5 gallon container) where [we] store drinking water. And [we] also have another bucket that is different from the other buckets because it has the valve on it. When everyone in the house sees that bucket, they know that this is drinking water. We have different containers for different uses.”	“We separate [the water]. Cooking is in one place, laundry in another, and drinking somewhere else. The drums outside are for gardening or having outside to throw water around. The cooking water is in the kitchen.” “One bucket is for collecting water from the source. Gadyen Dlo for drinking water from the community source. We don’t distinguish buckets. Only drinking water is separate.” “[We] store water for drinking separately. All other water is stored together—water for cooking, cleaning, bathing.”
**Bathing Practices**	“Not everybody needs to bathe at the same time, so we just have one bucket and we take turns. [I] also has a very small child who lives here, and [we] use a different type of bucket for the baby, it’s called a *kivet*.” “We have about 4 buckets for bathing. Each time we fill up the barrels with water and then put the water in the bucket for bathing when we need it.” “We share the buckets, it’s only one family. Unless you have someone that is very sick, then you would give this person their own bucket, but otherwise a family will share the buckets.”	“There is one bucket for my child to use for bathing, and I use treated water for that child’s bathing. [Me] and [my] husband use the same bucket for bathing and it is the water from the pump” “[We] empty out and refill the gallons between each use for each person. [We] use the same buckets but get new water.”
**Pregnancy Risks**	“You cannot get pregnant in the ocean, because of the salt in the water, the salty water gets inside your body and so you cannot get pregnant.” “I heard it’s impossible to get an infection or to get pregnant in the ocean because the water is very salty, and the ocean doesn’t keep the bacteria” “You can’t get pregnant because it’s salty. Some of my friends say you cannot get pregnant in the ocean, because after you have sex you can prevent pregnancy by drinking a lot of salt water so it’s the same thing as when you go to the ocean, because it’s salt water.”	“But I’ve heard you cannot get pregnant in the ocean, but I haven’t had the experience. I’m telling you what I’ve heard.” “You can't get pregnant in the ocean water. I have just heard that, but I don't know.” “You can’t get pregnant in the ocean, you can get pregnant in the pool. The ocean is a salty water and the pool is fresh water. The salt kills the sperm.”

### Damaged water source

Water sources being damaged or unobtainable emerged from the interviews mostly as a result of asking participants about their satisfaction with their water situation. However, there was a difference in what type of water source among the two sites. For example, in Gressier, most of the dissatisfaction with water sources came from the informal piping system not being functional. Participants described that either it cost too much money to install and have monthly payments, or they had it installed and it eventually stopped working. Among participants in Léogâne, complaints were primarily about the wells either being damaged, being too difficult to pump, or having a reddish discoloration in the water. Both sites have participants citing the additional burden and risk that comes with a broken or damaged water source. For instance, a participant describes in Table 1 the hardship that comes with having to cross National Road 2 to collect water from the water company. National Road 2 in Haiti is a very active highway where there is little regard for pedestrians, making safety an issue for anyone crossing it.

### Community involvement

Similar to damaged water sources, both sites had pockets of neighborhoods that shared an *esprit de corps* surrounding their water source–be it an informal piping system or well. This community bond with the water source resembled in unofficial cooperative wherein neighborhood members would either contribute by paying a monthly payment or pay to have the water source fixed if and when it became damaged. Specifically in Léogâne, 2–3 participants appeared somewhat resentful that there was no such commitment among their own neighbors to form a cooperative. As a result, these participants often spoke of the difficulties in having to travel to other neighborhoods to find a functioning water source when their own was broken.

### Treatment

Water treatment was discussed on a household scale within the socioecological framework, however, a large portion of participants from both populations also spoke about treatment at the community level as well. In this study, water treatment is defined as any method that can improve the quality of water for its intended use, be it cooking, bathing, or drinking. At the household level, it was fairly evenly split between participants at both sites whether or not they treated their water. Treatment options varied from acquiring free aquatabs from an NGO, buying aquatabs, using chlorine, or using a product called Gadyen Dlo, or water guardian. This product is a chlorine solution and is distributed throughout Haiti by Deep Springs International, UNICEF, and Jolivert Safe Water for Families [19]. In most cases when water was not treated, it was either because the water source was already perceived as treated and no additional treatment was necessary, or the participant simply did not treat it for various reasons such as lack of financial means. Specifically with drinking water, slightly more participants in Gressier used a pipe or well as their primary drinking source rather than purchasing drinking water; whereas in Léogâne, slightly more participants purchased their drinking water rather than using the well water. This study did not perform any water testing and cannot say with certainty which water was treated and which were not. It is also unknown how much of the water that is used as the primary drinking source is treated versus non-treated at the source itself (i.e. well or pipe). At the community level, several participants from Gressier identified ITECA (Institute of Technology and Animation), a non-governmental organization that supports areas of rural Haiti in agriculture, as a source for the piping system; however, there was dissatisfaction with this service since the water that is piped is not treated beforehand [20]. When asked who treats the water at the source directly, participants were unsure or could not say with certainty.

### Water separation & uses

For the most part, participants across both sites separate water into buckets dependent on the use of the water. For example, the participants from Gressier quoted in Table 2 indicate that Culligan gallons are used to store water for drinking. This is not uncommon since Culligan is a company based out of Haiti that sells water in various sizes and containers. In the case of the two participants quoted, what is known as a “Culligan gallon” is the five gallon container that Culligan sells. Additionally, when referring to the Culligan gallon container specifically, it is usually (in almost all cases from this study) a reused container used to gather and collect water from the water source, it is not necessarily bought each time. If bought, the water from any Culligan product is treated through chlorination, a sand filter, charcoal filter, softener, reverse osmosis, ultra violet, and ozonation processes [21].

With regards to water uses, participants from both communes used water collected from their water source for cooking, laundry, cleaning, and bathing. However, in Léogâne a small portion of participants indicated that when they use the water from their water source, specifically a well, they get a rash or skin infection. Many participants explained that water for bathing purposes should be boiled first, to avoid infection or disease.

### Bathing practices

Bathing practices was detached from the previous theme, water separation and uses, in order to capture if and how participants were sharing containers and/or reusing water for bathing purposes explicitly. There was variation between participants in both sites in regards to whether the same buckets were used, however, a majority of all participants stated they replace the water that had previously been used to bathe in. Very few stated that they treat the water they bathe in with any chemicals, such as chlorine, and rely primarily on boiling. Furthermore, even less participants shared that they clean the bucket(s) used for bathing between baths.

### Pregnancy risks

The risk of pregnancy reported by participants varied depending on the type of water. As indicated in Table 2, several participants from both communes share a belief that saltwater in the ocean prevents a woman from getting pregnant. While most participants in Gressier stated this openly, those in Léogâne hesitated and stated more than once in the interview that it was something they had heard from a friend or family member. Since both locations have easy access to the beaches on the northern part of the southern peninsula, this belief is particularly disturbing as it could result in passing sexually transmitted infections as well as unplanned pregnancy.

## Discussion

In several neighborhoods throughout Léogâne specifically, community members would describe either sharing the costs of maintenance of a well with neighbors or the opposite—feeling isolated and sometimes bitter because neighbors did not want to form an informal cooperative. As a result of wells being unregulated in Léogâne, this creates an unfortunate example of “The Tragedy of the Commons” paradigm, wherein individuals in certain neighborhoods act in their own self-interest instead of working together [22]. Cooperatives, specifically in Haiti, have been recommended for agriculture and watershed management [23–24]. Indigenous cooperatives provide additional support through co-financing and a social safety net to prevent against limited or no access to water [24]. Additionally, the International Labor Organization asserts that cooperatives have been found to be more inclusive and sustainable in countries with a more volatile governmental and offer resiliency during economic hardship [25]. Coffee farmers in Haiti have also been encouraged and financially supported by France’s Agency for Development to replicate Colombian coffee cooperatives to strengthen power among smallholder farms [26]. While water cooperatives in other developing countries have faced challenges with gender roles, voluntary work, water sources, and potential local instability; the benefits have included democratic local decision-making, open membership to widen the safety net and provide social capital, and strengthening their ability to have consistent access to a water source [27–30]. Therefore, these informal, microscale water cooperatives in Léogâne neighborhoods may prove beneficial for providing water security.

Access to drinking water is essential for the most aspects of living and is goal six of the Sustainable Development Goals which states clean water for drinking and sanitation should be accessible and affordable for all [31]. Water insecurity or deprivation has been linked to child mortality and life expectancy as well as poor health outcomes [32–34]. When water costs are inflated and/or water sources are located far from the household, this requires both time to obtain the water as well as the money or credit to purchase it [35]. Over 663 million people turn to fetching water from surface water sources, which is among the most unsafe drinking water, when neither of these precursors are available [35–36]. Even in areas where participants described their water source as improved, current literature supports that without testing the water there is no guarantee of microbial safety [37–40].

Water storage and container sharing differed between the two sites. For example, several participants in Léogâne described using the same bucket for collecting, cooking, washing clothes, and sometimes bathing. Whereas in Gressier, buckets were generally separated by use and water used for bathing was not reused for something or someone else. Despite those in Gressier relying largely on informal piped water systems, which may be slightly more safe than traveling for well water, these unregulated systems of water perpetuate a paradoxical arrangement that leaves the government unaccountable and can increase water insecurity in the future [41–43]. Conversely, since those in Léogâne must access their (well) water further than those in Gressier, there is likely to be more contamination from source to point-of-use [44]. Continued research regarding contamination of water at the point of use is needed to reduce the risks associated with this contamination of drinking water [45]. A study in rural India [46] indicated as much when fecal markers were found more frequently within a household water sources (45%) compared to public domains sources, such as ponds (8%) or groundwater (4%). While our study did not look specifically at storage practices within and around the home, major sources of contamination from fecal coliform and *Escherichia coli* occur after water collection, despite the water being high quality [47–50]. Reasons for contamination in drinking water within the household may be due to personal and domestic inadequate and unsanitary storage conditions [51]. Increased household water contamination poses a greater health risk on the individuals living there because it has the potential to bring new pathogens into the household that the residents may not have prior immunity to [52]. Similar studies urge the need for interventions at the household level, including water treatment, safe water handling, improved storage, and hand hygiene to ensure safe drinking water [53]. Subsequently, due to the ubiquitous nature of contamination within the household, further research should be done in Haiti to specifically look at domestic water storage to assess microbiological contamination.

While this study investigates a local phenomenon around water access and utility, due to its small sample size, it cannot be considered generalizable to the country as a whole, nor was that the intention. The sample for this study was derived from those surveyed with the HWISE survey, therefore, as with any survey, limitations exist from surveying those who were available and home during the hours enumerators conducted the survey. Additionally, despite having local Haitian translators translate in real time and re-listen to recordings to ensure translations were correct, there is always the potential for nuanced linguistic meaning that is lost through translation.

## Conclusion

This study explored the knowledge, attitudes, and practices around water insecurity in urban and peri-urban Haiti to better understand the issue. Results have shown that there are several culturally based perceptions surrounding water and illness, specifically related to reproductive health. This gap in knowledge opens the door for future interventions to focus on tackling common misconceptions with the spread of disease and what role water can play in preventing and contracting disease.

Researchers are currently working with local partners (NGOs, faith-based organizations, and health clinics), and encourage others seeking to work in this region, to focus on preventative measures with regards to waterborne diseases as a result of water insecurity. A study done in Bangladesh [54] around water insecurity reminds us that despite foreign efforts, be it through humanitarian aid or research, there are still an indiscernible amount of people that face water insecurity that severely effects their health and livelihoods. Furthermore, successful models of preventing water insecurity in Haiti should be fostered. One successful example includes the Gift of Water where resources (buckets for purifying water) paired with training (around water, sanitation, and hygiene as well as how the buckets should be utilized) have shown improvement in access to safe water and reduced the risk of disease transmission [55]. Therefore, community-based NGOs such as this should be replicated and/or scaled up throughout the country (with appropriate tailoring for local needs) to benefit communities by reducing water-related morbidity.
